# Effects of nickel content on the microstructure, microhardness and corrosion behavior of high-entropy AlCoCrFeNix alloys

**DOI:** 10.1038/s41598-020-78108-5

**Published:** 2020-12-03

**Authors:** M. López Ríos, P. P. Socorro Perdomo, I. Voiculescu, V. Geanta, V. Crăciun, I. Boerasu, J. C. Mirza Rosca

**Affiliations:** 1 Mechanical Engineering Department, University of Las Palmas de Gran Canaria, Campus Universitario Tafira, Edif.Ingenieria, 35017 Gran Canaria, Spain; 2 Faculty of Industrial Engineering and Robotics, Politehnica University of Bucharest, 313 Splaiul Independentei, 060042 Bucharest, Romania; 3 Faculty of Materials Science and Engineering, Politehnica University of Bucharest, 313 Splaiul Independentei, 060042 Bucharest, Romania; 4 National Institute for Laser, Plasma and Radiation Physic, Magurele, Romania; 5 Extreme Light Infrastructure-Nuclear Physics, IFIN-HH, Magurele, Romania

**Keywords:** Chemistry, Materials science

## Abstract

In this study the effect of three different nickel concentration on the microstructure, hardness and corrosion properties of high entropy alloys (HEAs) from AlCrFeCoNi system as an alternative material for medical instruments fabrication was investigated. The analyzed HEAs were AlCrFeCoNix obtained by vacuum arc remelting from high purity raw materials and having nickel atomic ratio x = 1.0, 1.4 and 1.8. The microscopy examination revealed the dendritic morphology for the reference alloy (AlCrFeCoNi) and that the extent of the interdendritic areas increased with the concentration of nickel while Cr was more segregated in the interdendritic areas than in dendrites. Hardness values decreased as the percentage of nickel increased due to the dissolution of the precipitates in a nickel-rich matrix and consequently the formation of continuous solid solutions. The corrosion properties of the synthesized HEAs were evaluated using a potentiodynamic polarization method. The alloys were immersed in Simulated Body Fluid during one week and the corrosion parameters were recorded. The low corrosion rates, low corrosion currents and high polarization resistance attest the good stability of these HEAs in simulated biological environment indicating their possible use for surgical and dental instruments.

## Introduction

Classic metallic alloys generally contain one metal in a high proportion, called the base metal, and very rarely two metals in similar proportions. Although small amounts of other elements are added, this can make a big difference in the characteristics of the obtained alloy. Due to the great advance of science and technology, new metallic alloys containing more than 2 base metals, with a different metallurgical concept, have been recently explored^1,2^.

High-entropy alloys (HEAs) are one of the most promising results of the exploration of new chemical compositions for metallic materials with improved performance^3–5^. Originally, they were defined as an alloy with at least five metallic elements with atomic percentage between 5 and 35%^6^.

One of the basic alloy from HEA category, AlCoCrFeNi, was discovered in 2014 by Zhang’s group at University of Science and Technology from Beijing, China^2^. Many other groups have joined the research effort to understand this HEA microstructure^7,8^, hardness^9,10^, strength^11,12^, friction and wear^13^ and thermal resistance^14,15^ particular properties. Although many interesting topics have been explored, only few studies deal with corrosion properties of this high-entropy alloy, in general depending of fabrication method: if is synthesized by laser additive^16^, by electrospark process^17^ and by spark plasma sintering with pre-alloy powders obtained through gas atomization^18^. Other studies, with different aluminium concentration were performed^19–21^.

In the late last century, the progress of materials science led to the rapid development of biomedical materials. Nowadays, titanium alloys are widely used as implants and prosthesis in the human body because of their excellent biocompatibility and low density. However, titanium alloys do not have sufficient strength characteristics for medical instruments used for surgery or prosthetic devices. The recent development of HEAs provides a new generation of biomaterials which may can be used for medical devices.

To be able to use the high-entropy alloys for the manufacturing of medical instruments (like cutters, saws, scalpels etc.), their mechanical characteristics and corrosion resistance in physiological fluids which contain 1% wt. NaCl, must be tested. Furthermore, if the material corrodes due to chemical attack, some corrosion products that will form can produce undesirable reactions at metal-tissue interface. To avoid this deleterious effect, the chemical composition of the new alloys must be carefully designed^22^. The material needs to be inert in contact with the human body, so it won´t cause any metal contamination when used internally. Together with the mechanical properties^23,24^, corrosion resistance plays a critical role in determining the successful use of HEAs for biomedical applications^25,26^. In this study the effect of three different nickel concentrations on the microstructure, hardness and corrosion properties of high entropy alloys from AlCrFeCoNi system has been investigated. The reason for adding nickel is that nickel generally increases ductility and hardness. Nickel improves heat treatment properties by expanding the critical temperature level, it does not form oxides and this increases strength without decreasing ductility. The results presented below indicate that the structure and corrosion behavior of AlCrFeCoNix alloys strongly depends on the Ni content.

## Experimental

### Materials and samples preparation

The high entropy AlCrFeCoNix alloys (with x = 1.0, 1.4 and 1.8) were obtained in the ERAMET Laboratory of the Politehnica University of Bucharest, using the MRF ABJ 900 Vacuum Arc Remelting (VAR) installation^10,25^. The theoretical degree of assimilation of the chemical elements during melting and the possible losses by vaporization were taken into account for designing the metallic charge. Highly pure raw materials, including Al, Cr, Fe, Co and Ni (at least 99.5%) were used. In order to obtain the adequate homogeneity, the obtained alloys were flipped and re-melted in VAR equipment for 6 times (3 times on each part) under inert atmosphere of Argon.

Samples in the form of rods of about 10 cm long and 1 cm diameter were obtained. The rods were transversally cut and some samples were selected for homogenization (annealing to 1100 °C for 48 h followed by water quenching). For structural, compositional and mechanical analyses the samples were embedded into an epoxy resin cylinder and then their surface was prepared in 3 stages: (1) polishing with SiC abrasive papers of progressive grain size from 240 to 2000 grit; (2) final polishing with 0.1 µm alpha alumina paste; (3) cleaning in ultrasonic deionized water.

### Test environment

All the measurements were performed in Ringer Grifols solution (from Grifols Laboratories, Barcelona, Spain) with the following composition in mmol/l: Na^+^ 129.9; K^+^ 5.4; Ca^2+^ 1.8; Cl^−^ 111.7 and C_3_H_5_O_3_^−^ 27.2. It is a modified physiological solution in which part of the sodium ions are replaced by calcium and potassium ions and parts of the chlorine ions by lactate ions. The lactate ions are transformed into bicarbonate ions allowing a regulation of the solution pH. The tests were conducted at 37 ± 0.1 °C in a thermostatic bath.

### Microstructural characterization

To study the microstructure of the alloys by optical microscopy, their surface was etched by electrochemical route using a solution of 10% oxalic acid, a current of 5 A and an immersion time of 4 s. The observations of the surface were made using an OLYMPUS PME 3-ADL microscope.

Scanning electron microscope (SEM) observations were made using a S LoVac of the Apreo Field Emission Scanning Electron Microscope (THERMO FISHER SCIENTIFIC, Co., USA) equipped with a TEAM EDX spectrometer. For ensuring the best high vacuum imaging and analytic conditions the microscope was set to run at 20 kV voltage and 1.6 nA beam current, for working distance of 10.0 mm.

### Electrochemical measurements

The electrochemical measurements were made with a conventional three-electrode electrochemical cell: the sample as working electrode, Pt as counter electrode and a saturated calomel electrode (SCE) as reference electrode. The used potentiostate was a SP-150 (BioLOGIC Science Instruments) controlled by a computer with EC-LAB software package.

#### Open circuit potential (OCP)

Open circuit potential measurements during 3 days were performed, followed by potentiodynamic polarization measurements. All tests were performed three times and data were processed using EC-LAB software.

#### Potentiodynamic polarization studies—polarization resistance and Tafel slopes

In order to calculate the Tafel slopes for the partial anodic processes (b_a_), and the Tafel slopes for the partial cathodic processes (b_c_), the linear polarization curves have been shifted from E_OCP_ − 150 mV to E_OCP_ + 150 mV using a scanning rate of 10 mV/s. The polarization studies to evaluate the passivation process continued with measurements from − 800 mV (vs.SCE) to + 500 mV (vs.SCE), increasing the potential at a scanning rate of 1 mV/s. SP-150 potentiostate was used to perform the tests and data were processed using EC-LAB software, both from BioLOGIC Science Instruments. Results showed the potentiodynamic polarization curves and the breakdown potential.

### Microhardness measurements

The HEAs Vickers microhardness has been measured by an indentation test using a REMET HX-1000 Microhardness Tester. The samples, with the surfaces polished to mirror quality for good vision of the prints, were indented every 0.5 mm along the diameter. The tests were carried out according to the regulation UNE-EN ISO 6507-1:2006, applying a load of 100 g during 15 s. A minimum of 5 indentations were made on each sample and the average value was calculated, expressing it as the Vickers hardness (HV).

### XRD analysis

X-ray diffraction experiments were performed with the aid of an empyrean diffractometer (MALVERN-PANALYTICAL). The instrument was working with a Cu Kα anode at a power of 45 kV and 40 mA in the Bragg–Brentano geometry. The samples were rotated during acquisition to ensure a better data collection. The acquired patterns were simulated to extract the crystalline phase present, lattice parameter and grain size with the aid of HighScore Plus software from MALVERN-PANALYTICAL.

## Results and discussions

### Microstructure

The phase structure of an alloy is critical for its biocompatibility and depends on the solubility of the alloying elements. The interaction between the phase structure and the biologic environment determines which elements will be released and, therefore, how the body will respond to the alloy. The grain size affects the corrosion processes because the grain boundaries influence the corrosion behaviour. The smaller the grain size of the samples, the higher the critical current density they will have as the edges of the grains store internal energy that promotes the corrosion^27^.

**Figure 1:**
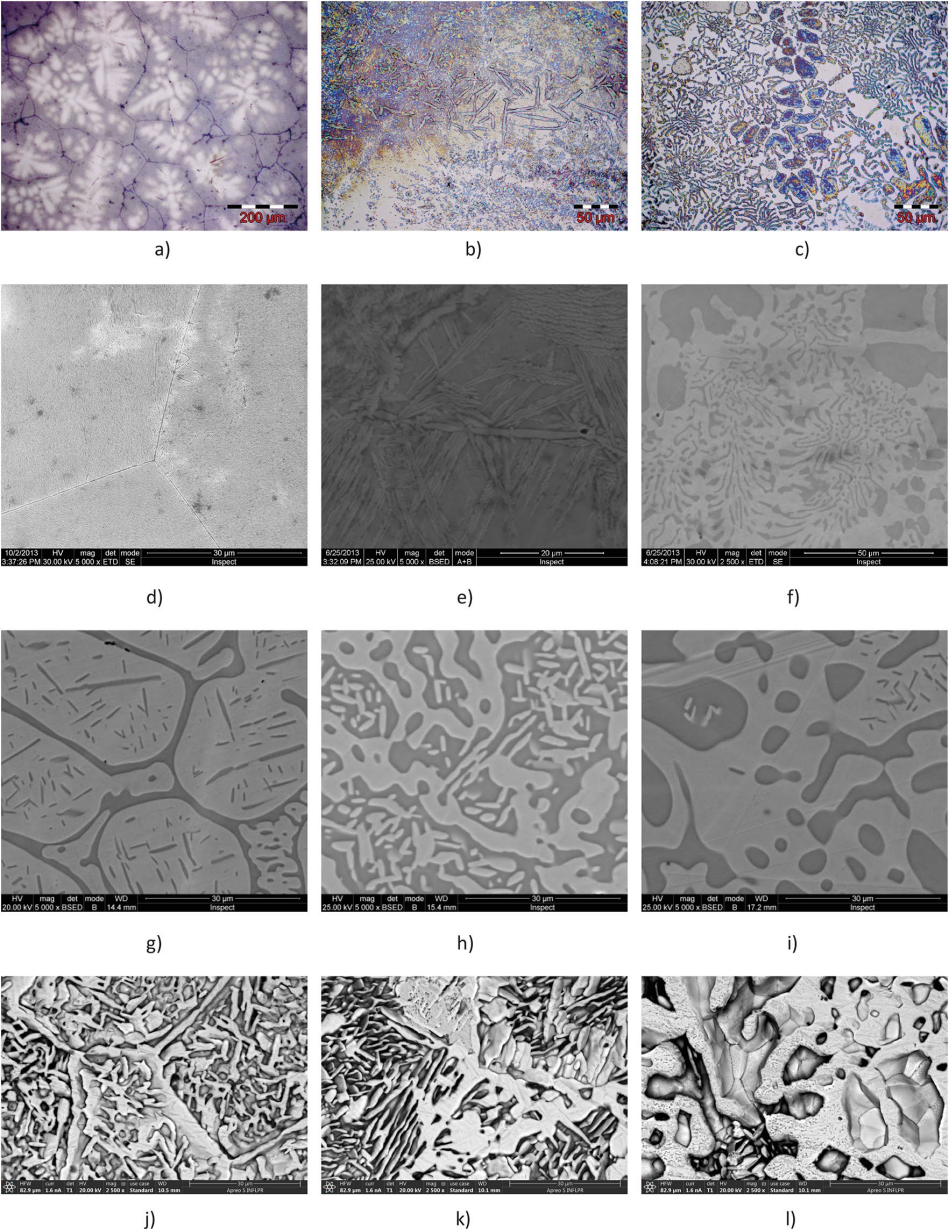
Microstructure evolution of HEAs (Ni_1_, Ni_1.4_ and Ni_1.8_) during thermal and chemical processing: (**a**), (**b**), (**c**) optical microstructure; (**d**), (**e**), (**f**) SEM as-cast microstructure; (**g**), (**h**), (**i**) SEM heat treated microstructure; (**j**), (**k**), (**l**) Thermal treated and corroded microstructure.

The microstructures of the analysed HEAs before corrosion tests are shown in Fig. 1a–f. The overall look of the optical microstructures is dendritic (Fig. 1a–c). The different concentrations of the alloying elements involves the morphology of the phases. Thus, in the case of AlCrFeCoNi the aspect of the dendrites is quite round, while in the AlCrFeCoNi_1.4_ sample we can observe needle forms that are oriented in different directions. The AlCrFeCoNi_1.8_ sample combines rounded phases with needle-like phases, consistent with the observation reported by Cao et al.^28^.

Chrome induces the formation of a protective and compact oxide layer on the surface of nickel alloys, the optimum corrosion resistance being obtained with Cr contents of about 16–27%. If the Cr content is lower, the alloy may not be able to develop a passive film adequate for a good corrosion resistance. AlCrFeCoNi has a spinodal structure quite typical for high entropy alloys, as we reported before^5^. This structure determines the smaller dimension of the phases and higher interfaces area increasing the hardness value.

The SEM observations are presented in Fig. 1d–f. The microstructural aspect is similar to that observed by optical microscopy. In the case of Ni_1_ alloy, the crystalline grains with crystallites are observed, organized like Chinese letters, bordered by linear limits (Fig. 1d). As the Ni content increases in the Ni_1.4_ alloy, the appearance of acicular phases is observed (Fig. 1e). As further increase of nickel content in Ni_1.8_ alloy the acicular phases become rounded (Fig. 1f).

The elements Al, Co and Ni can form continuous solid solutions with the same composition in the matrix of dendritic and interdendritic zones while Cr and Fe segregated more in the spherical precipitates of dendritic region^5,7^. This suggests that the partitioning of elements from the solution phase of HEA is inherently related to the enthalpy and miscibility between the various atoms present^22^.

The heat treatment performed after casting promoted homogenization of chemical composition and changes of microstructure aspect (Fig. 1g–i). The linear appearance of the grain boundaries was replaced by curved connections and the amount of needle phase decreased. In Fig. 1g three types of phases can be distinguished, as follows: a majority phase (light gray) with dendritic appearance, an inter-dendritic phase (dark gray) and thin needle like phase formed inside the majority phase. As the nickel concentration increased (Fig. 1h,i), there was a compositional change of the 2 major phases (dark gray and light gray) and a decreasing of the needle-like phase number.

The surface of the samples was examined also after performing heat treatment and corrosion test. For all the samples, pitting corrosion was observed. The images of the corroded surfaces highlight the acicular-looking phases that formed in the microstructure after the heat treatment (see Fig. 1j–l). It is observed how the chemical solution partially dissolved the surface film and preferentially attacked the alloy phases. As the Ni content in sample Ni_1.8_ increases to 31 at.% (Fig. 1l), more extended corrosion effects on the alloy phases are observed.

### EDS analysis

The EDS analyses have been performed on micro-zones, having the same square area, see Fig. 1g–l. The results of the chemical composition for the three alloys are presented in Table 1.

**Table 1:** EDS global analyses on micro areas for HEAs after different processing stages.

Element	Ni_1_			Ni_1.4_			Ni_1.8_		
	Corr	TT	T + C	Corr	TT	T + C	Corr	TT	T + C
Al, at%	**12.00**	**9.89**	**2.57**	**11.68**	**14.28**	**3.03**	**10.59**	**10.46**	**2.77**
wt%	6.33	5.14	1.29	6.12	7.54	1.52	5.47	5.37	1.38
Error,%	5.47	5.43	4.13	2.84	5.47	5.74	2.92	5.82	5.76
Co, at%	**6.59**	**8.48**	**21.85**	**3.75**	**7.40**	**21.49**	**3.34**	**5.36**	**20.49**
wt%	7.58	9.62	24.07	4.29	8.54	23.46	3.77	6.01	22.33
Error,%	5.34	5.02	1.79	3.32	4.43	1.79	3.88	4.51	1.76
Cr, at%	**28.72**	**26.98**	**31.83**	**32.61**	**22.67**	**28.51**	**29.62**	**22.95**	**26.16**
wt%	29.17	27.01	30.93	32.93	23.07	27.46	29.50	22.71	25.15
Error, %	1.93	1.90	1.90	1.99	2.00	1.95	2.04	2.11	1.97
Fe, at%	**26.56**	**26.40**	**26.41**	**24.75**	**21.52**	**23.52**	**22.94**	**21.39**	**21.07**
wt%	28.98	28.38	27.57	26.85	23.53	24.33	24.54	22.73	21.76
Error,%	2.05	1.95	1.87	1.81	2.11	1.94	1.90	2.30	1.99
Ni, at%	**23.28**	**24.99**	**12.73**	**24.78**	**31.13**	**19.62**	**31.33**	**37.68**	**25.14**
wt%	26.58	28.24	13.97	28.26	35.78	21.33	35.24	42.10	27.29
Error,%	2.12	1.94	2.05	1.73	2.11	1.93	1.67	1.98	1.85
O, at%	**2.38**	**2.46**	**3.54**	**1.39**	**2.22**	**2.77**	**1.08**	**1.61**	**3.25**
wt%	0.74	0.76	1.06	0.43	0.70	0.82	0.33	0.49	0.96
Error, %	5.15	4.57	4.13	2.78	5.21	4.40	2.73	6.24	4.28

Corr, corroded; TT, Thermal treated; T + C, Thermal treated and corroded.

Analyzing the EDS results from Table 1 it is observed that Al concentration decreases in all samples that were simultaneously heat treated and corroded, from about 10 at% to 2.57–3.03 at%. A similar evolution is observed in terms of Ni concentration, which decreases from the maximum values existing in the heat treated samples (24.99 at% for Ni_1_ to 37.68 at% for Ni_1.8_) to just over half of the initial values (12.73 at % Ni for Ni_1_ to 25.14 at% Ni for Ni_1.8_). Cr and Fe maintain their concentrations within tight limits (maximum variations of 5 at%), and Co records the largest increases in concentration for simultaneously heat-treated and corroded samples. This behavior emphasizes the high chemical stability of Co in the metal matrix of the analyzed alloys.

**Figure 2:**
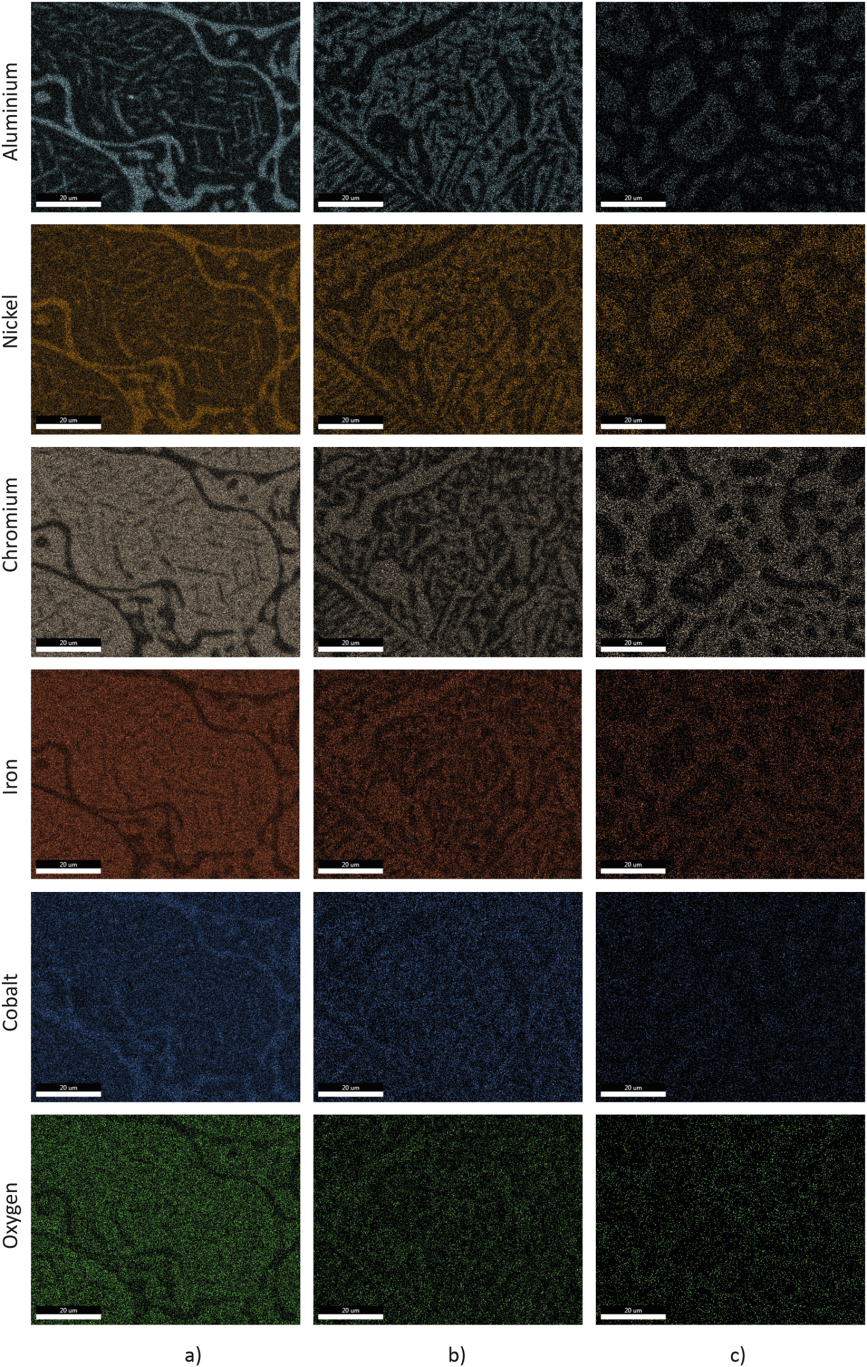
Atoms distribution of main elements of HEAs micro-area. (**a**) in Ni_1_ TT sample; (**b**) Ni_1.4_ TT sample; (**c**) Ni_1.8_ TT sample.

Based on the EDS analysis performed on different phases, following the initial values of Co and Cr content in those phases, the specific tendency of segregation and association of these elements to form common phases is observed on atoms distribution maps (see Fig. 2).

In the case of Ni_1_ (AlCrFeCoNi) TT sample, the chemical microanalysis on light grey phase indicates that it contains about 5.08 at% Al, 9.14 at% Co, 31.76 at% Cr, 29.80 at% Fe and 20.51 at% Ni. The dark grey phase contains 27 at % Al, 8.55 at% Co, 10.37 at% Cr, 14.15at% Fe and 38.26 at% Ni.

Regarding Ni_1.4_ (AlCrFeCoNi_1.4_) TT sample, the light grey phase contains 4.40 at % Al, 7.16 at % Co, 34.18 at % Cr, 28.96 at % Fe and 21.42% Ni. The dark grey phase contains 23.53 at% Al, 7.21 at % Co, 13.35 at % Cr, 15.21 at % Fe and 39.23 at % Ni.

Finally, in the case of Ni_1.8_ (AlCrFeCoNi_1.8_) TT sample, the light grey phase contains 21.73 at% Al, 5.31 at %Co, 9.85 at% Cr, 12.82 at% Fe and 48.88 at% Ni. The dark grey phase contains 5.51 at % Al, 6.02 at % Co, 29.06 at% Cr, 24.98 at.% Fe and 31.01at % Ni.

These results highlight the tendency of Al and Ni to form stable compounds (dark grey phase) and quite equal distribution of the other elements in light grey phase or in the acicular phases.

### X-ray diffraction

**Figure 3:**
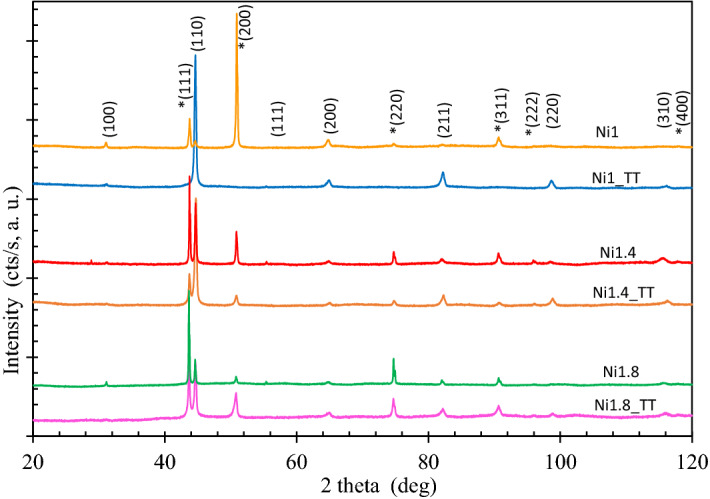
XRD patterns acquired from as-cast samples and from thermal treated samples (Miller indices for the new FCC phase induced by a higher Ni content are marked by symbol #).

The XRD patterns acquired from as-cast samples are displayed in Fig. 3. The Ni_1_ sample exhibited a pure primitive cubic phase (space group Pm-3m, number 221), matched very well by reference pattern 04-018-5047, Al0.4Co0.4Cr0.4Fe0.4Ni0.4. Increasing the Ni content resulted in the appearance of a new FCC phase (Fm-3m, group 225, a = 3.5643 Å), that was matched by reference pattern 04-022-2301, Co_0.25_Cr0.25Fe_0.25_Ni_0.25_ and indicated by symbol # in front of the Miller indices in Fig. 3. Also, Al_0.9_Ni_4.22_ (pattern 00-050-1294) with a slightly different lattice parameter (FCC, a = 3.5700 Å) and Cr_0.10_Fe_0.65_Ni_0.25_ (pattern 04-019-2390) with a slightly different lattice parameter (FCC, a = 3.5920 Å) are good matches. The presence of an Al-Ni FCC type compound after the thermal treatment is strongly supported by the EDS results. After corrosion treatment, a strong decrease in Ni and Al concentrations was observed, which was probably caused by the dissolution of this compound, while the others elements were not that much affected. SEM images of the corroded surface also suggest that a phase initially present was dissolved and disappeared from the surface region.

XRD patterns acquired from samples after the thermal treatment exhibited a mixture of two cubic phases: a primitive one and a FCC one (Fig. 3), with the relative percentage displayed in Table 2, although the presence of other FCC type compounds as those mentioned above could not be ruled out. The patterns also displayed narrower diffraction peaks, indicative of grain growth. The lattice parameters of the Pm-3 m and FCC phases, also displayed in Table 2 did not significantly changed with the increase of Ni content or the thermal treatment.

**Table 2:** Samples phase composition, lattice parameters and grain size.

Sample	Pm-3m (PDF 04-018-5047)			Fm-3m (PDF 04-022-2301)		
	Content (%)	Lattice parameter (Å)	Grain size (Å)	Content (%)	Lattice parameter (Å)	Grain size (Å)
Ni_1_	100	2.876	398	–	–	–
Ni_1.4_	78	2.870	414	22	3.595	327
Ni_1.8_	37	2.873	365	63	3.599	402
Ni_1__TT	16	2.875	574	84	3.588	562
Ni_1.4__TT	73	2.871	937	27	3.591	1813
Ni_1.8__TT	75	2.872	1258	25	3.591	1259

With the increasing of nickel concentration, the lattice parameter of the primitive cubic phase varies very slightly, from 2.876 Å to 2.870 Å, while the grain size remains almost the same (from 398 to 414 and 365 Å) for as-cast samples. For the FCC phase, the lattice parameters also vary very slightly but the grain size increases with the nickel content for both as-cast andannealed samples. It can be seen that, for all the studied alloys, the lattice parameter of the two phases varies marginally, which was also reported for other HEAs^29^.

The results show a good homogeneity of the samples with two main solid solutions formed and some minor compounds segregating in the dendritic zone.

After the thermal treatment, the elemental composition of HEAs does not change; however, after the corrosion stage, the Al concentration in the surface region significantly dropped, followed by Ni; Co concentration went up, while Fe and Cr did not change much.

To explain these results, the formation after the thermal treatment of an Al-Ni compound is hypothesized, which should be corroded faster than the main HEA phase. There are several Al-Ni compounds having an FCC lattice and a lattice parameter very close to that of FCC AlCoCrFeNi HEA that could explain the results.

### Open circuit potential (OCP)

**Figure 4:**
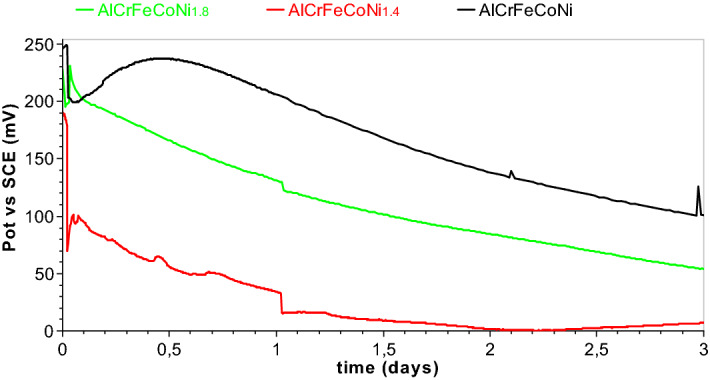
Variation of open circuit potential with time for the three alloys (AlCrFeCoNi, AlCrFeCoNi_1.4_, AlCrFeCoNi_1.8_) in Ringer solution.

Open circuit potential measurement curves during one-week immersion are shown in Fig. 4.

For all analyzed HEAs, after a short immersion time of about some hours, there is an increase in the corrosion potential due to the growth of passive layers on the surface of the alloys. During the first 24 h, the OCP for all the three alloys increases with 30–50 mV due to the build-up of the passive layers on the surface of the HEAs. The maximum value of OCP is 236 mV for AlCrFeCoNi and 235 AlCrFeCoNi_1.8_ and almost half of this value, 102 mV, for AlCrFeCoNi_1.4._

After the OCP reaches the maximum value, it begins to decrease. This decrease is due to the changes in the characteristics of the surface film.

From the curves can be seen continuous breakages and repairs of the passive layer.

### Potentiodynamic polarization result

Polarization techniques have been used respecting the indications of ASTM Subcommittee G01.11 on Electrochemical Measurements in Corrosion Testing regarding the reproducibility of cyclic potentiodynamic polarization measurements for determining the susceptibility to localized corrosion^30^.

**Figure 5:**
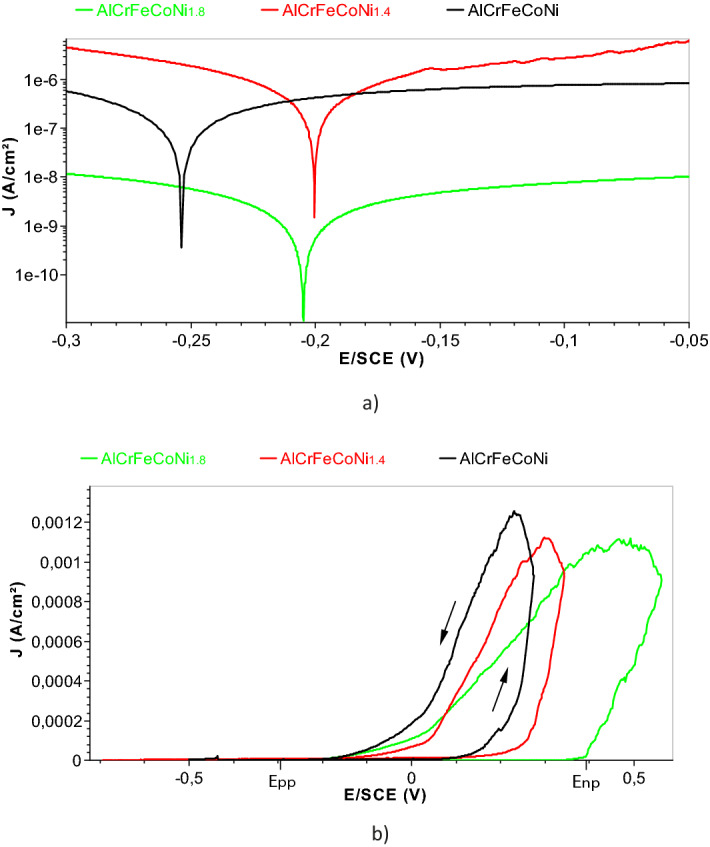
(**a**) Tafel curves for AlCrFeCoNi_1_, AlCrFeCoNi_1.4_ and AlCrFeCoNi_1.8_ after one-week immersion. (**b**) Potentiodynamic polarization curves for AlCrFeCoNi, AlCrFeCoNi_1.4_, AlCrFeCoNi_1.8_ presented in linear axis in order to reveal the nucleation pitting potential and pitting protection potential).
  The values of E_corr_, i_corr_, b_a_, b_c_ and V_corr_ were determined by EC-Lab software and presented in Table 3. An alloy will corrode if b_c_ is greater than b_a_ and will be subjected to passivity if b_c_ is smaller than b_a_. The higher values of b_a_ vs b_c_ for all the three alloys indicates an anodic control of the corrosion process which implies the existence of a passive layer on the sample’s surface. The point resulted in the intersection of the two Tafel slopes has the coordinates i_corr_ and ZCP (zero current potential). It can be seen that in all the cases, the difference between OCP and ZCP is only few mV. This indicates that the errors introduced into the values of kinetic parameters are negligible with the variations of the charging current.

1. Plots in a semi-logarithmic version between − 150 mV (vs OCP) and + 150 mV (vs OCP) after 1 week in Ringer solution are displayed (see Fig. 5a).

**Table 3:** Electrochemical parameters of corrosion process estimated through Tafel approximation.

Alloy	E_corr_ (mV)	i_corr_ (nA/cm^2^)	b_a_ (mV)	b_c_ (mV)	Corrosion rate (mmpy)
AlCrFeCoNi	− 255.59	428	638.4	255.1	5.92E−03
AlCrFeCoNi_1.4_	− 199.7	748	169.2	124.5	3.00E−02
AlCrFeCoNi_1.8_	− 206.86	374	347	185.8	1.25E−02
  The corrosion current densities obtained for the analyzed HEAs are much lower than that of the 304SS^31^, which means that these HEAs are more resistant to general corrosion than 304SS.
  With the increase of Ni content, the corrosion rate of the alloy presents a non-linear trend mainly due to the fact that Ni content is not the only factor affecting the corrosion process (for example: microstructure, element distribution, etc.).
2. Pitting potential
  The pitting potential (or pit nucleation potential E_np_) is one of the most important parameter characterizing the susceptibility of HEA to pitting corrosion. It is the potential at which the passive film formed on the HEA surface is damaged and the current density begins to increase drastically in the passive range due to the pits nucleations. Plots in a linear version of I vs E between − 0.8 V and + 0.5 V vs Ref (see Fig. 5b) were performed in order to determine the pit nucleation potential (pitting potential) and pitting protection potential (the potential in the reverse scan associated with a drop in current density caused by the repasivation of pits).
  A scan rate of 1 mV/s was used and considered to be sufficiently slow to prevent any distortion of the curves. The values of nucleation pitting potential were 185 mV for AlCrFeCoNi, 245 mV for AlCrFeCoNi_1.4_ and 385 mV for AlCrFeCoNi_1.8_. The obtained values characterize the resistance of the analyzed HEA to pitting corrosion and can therefore be considered a measure of the susceptibility of HEA in simulated body fluid. AlCrFeCoNi_1.8_ has the most positive pit nucleation potential: the more positive Enp, the more resistant the alloy is to pitting.
  Among the three tested alloys, the difference between E_np_ and E_pp_ for AlCrFeCoNi_1.8_ is the largest, which suggests that the repasivation tendency of pits on this alloy is large and only at higher potential these pits can transform to stable pits.

### Microhardness

Five indentations have been made for each sample and the average value was calculated. The hardness values decrease with the increase of nickel concentration (562HV for AlCrFeCoNi, 455HV for AlCrFeCoNi_1.4_ and 316HV for AlCrFeCoNi_1.8_) as a result of dissolution of Cr and Fe precipitates in the nickel-rich matrix, forming a stable solid solution as we reported before^10^. For other HEA system, substitution of Al with different Zr concentrations, determine changes in the microhardness values, attributed to the phase changes in the alloy structure^23^.

As has been demonstrated in a few studies^11,24^ most metals and alloys exhibit strengthening effect by grain refinement due to the boundaries that function as impediments to dislocations. The decreasing of grain boundary density lead to the microhardness (and equivalent yield strength) values decreasing in HEAs which is consistent with the concept that the lattice of the crystal is seriously distorted and dislocations movement is more difficult than in conventional alloys^32,33^.

## Conclusions

In this study, the effects of nickel content on the microstructure, microhardness and corrosion behavior of high-entropy entropy AlCoCrFeNi_x_ alloys in simulated body fluid were investigated and following conclusions were drawn:

1. The microscopy examination revealed the dendritic morphology for as cast alloy AlCrFeCoNi_1.0_ and the increase of the extent of the interdendritic areas by increasing the nickel concentration for AlCrFeCoNi_1.4_ and AlCrFeCoNi_1.8_. The annealing determined a more uniform distribution of the phases for the three high-entropy alloys and the modification of the morphology of the grain boundaries. It also resulted in a significant increase of the grain sizes.
2. The formation after the annealing treatment of an Al-Ni compound is hypothesized, which should be corroded faster than the main HEA phase. There are several Al-Ni compounds having an FCC lattice and a lattice parameter very close to that of FCC AlCoCrFeNi.
3. The low corrosion rates and low corrosion currents demonstrate the good stability of the studied samples of AlCoCrFeNi_x_ (x = 1.0, 1.4 and 1.8) in simulated biological environment.
4. The lattice parameter of the cubic phase varies very slightly, from 2876 to 2870 Å by increasing the Ni content, but the grain size decreases considerably (from 398 to 206 Å) for as-cast samples. For the FCC phase, the lattice parameters also vary very slightly but the grain size increases with the nickel content for both as-cast and annealing samples.
5. The results proved that by manipulating the composition and structure of HEAs their mechanical and chemical performance could be optimized to meet the requirements for their usage as novel medical instruments materials.

## Data Availability

The datasets generated during the current study are available from the corresponding author on reasonable request.
